# A Deep Learning Framework for EEG-Based Decoding of Visually Imagined Arrows with Different Colors and Directions

**DOI:** 10.3390/bios16070383

**Published:** 2026-07-14

**Authors:** Rami Alazrai, Oula Hatahet, Sahar Qaadan, Youssef Alothman, Mohamed Bader-El-Den

**Affiliations:** 1 School of Computing, German Jordanian University, Amman 11180, Jordan; oula.hatahet@gju.edu.jo; 2 College of Computer and Systems Engineering, Abdullah Al Salem University, Block 3, Khaldiya 72303, Kuwait; mohamed.baderelden@aasu.edu.kw; 3 School of Applied Technical Sciences, German Jordanian University, Amman 11180, Jordan; sahar.qadan@gju.edu.jo; 4 School of Computing, University of Portsmouth, Portsmouth PO1 3HE, UK; youssef.alothman@port.ac.uk

**Keywords:** electroencephalography, brain–computer interface, neuroscience, time–frequency analysis, convolutional neural network, visual imagery, assistive technology, good health and well-being

## Abstract

Brain–computer interface (BCI) systems have demonstrated significant potential across medical, educational, and entertainment domains. Recently, visual imagery (VI) has emerged as an alternative to traditional motor imagery (MI) paradigms, offering a broader spectrum of control signals for dexterous assistive devices. In this study, we propose a novel BCI framework for classifying visually imagined arrows defined by different colors and directions. The proposed framework employs the Choi–Williams time–frequency distribution (CW-TFD) to construct a joint time–frequency–spatial representation (TFSR) of EEG signals. The resulting TFSR is converted into grayscale images and provided as input to a newly designed convolutional neural network (CNN), which performs 16-class decoding of visually imagined arrows defined by combined color and direction attributes. A new EEG dataset was collected from 16 subjects who imagined 16 distinct arrows comprising four colors and four directions. The framework achieved an average classification accuracy of 95.05% and a Cohen’s kappa score of 0.947 across the 16 classes. To comprehensively evaluate the proposed approach, three comparative analyses were conducted. First, multiple time–frequency representations were assessed for VI-based EEG decoding. Second, the proposed CNN architecture was benchmarked against several state-of-the-art pre-trained deep learning models. Third, the framework was compared with conventional machine learning classifiers using handcrafted features. Results demonstrate that the constructed CWD-based TFSR combined with the proposed CNN consistently outperforms alternative representations and classification models. These findings demonstrate the feasibility of decoding an expanded set of visually imagined color–direction arrow commands in a subject-specific EEG-based BCI setting, supporting further development of calibrated VI-based BCI systems for assistive and interactive applications.

## 1. Introduction

Communication is a fundamental human need whose demand and complexity continue to grow. In particular, technology-driven human–computer interaction has opened numerous opportunities in medical, entertainment, and military domains [1]. Brain–computer interface (BCI) systems have emerged as a promising technology for facilitating and enhancing communication in such application areas. A BCI system integrates software and hardware components that enable users to operate and interact with external devices directly through brain activity [2,3].

In the medical domain, BCIs can significantly benefit individuals with neurological or physical impairments that hinder their ability to communicate and interact effectively with their environment, such as patients in paralyzed or locked-in states [4]. In the gaming and entertainment industries, BCIs can increase user engagement and foster the development of innovative interactive products [5]. Moreover, advanced BCIs can expand applications in virtual reality (VR), enabling users to control virtual environments through a broader range of mental commands [6,7].

Several neuroimaging modalities have been employed to record brain activity, including electroencephalography (EEG) [8], positron emission tomography (PET) [9], and functional magnetic resonance imaging (fMRI) [10]. Among these techniques, EEG is particularly attractive due to its portability, affordability, non-invasive nature, and high temporal resolution [11], making it well suited for real-world BCI applications [12,13].

Over the past two decades, most BCI systems have relied on motor imagery (MI) paradigms to enhance control and communication in both virtual and physical environments [4,14,15]. In MI-based BCIs, users imagine limb movements without physically executing them. For amputees or stroke patients, this may involve imagining movement of a missing or paralyzed limb [12,13,16,17,18,19,20,21]. However, MI-based systems are often constrained by the limited number of distinct control signals that can be reliably extracted. Furthermore, many users experience difficulty performing pure motor imagery and tend instead to form visual representations of movements rather than engage in actual motor simulation [4]. These limitations restrict the scalability of MI-based BCIs for controlling complex assistive devices that require a richer command set [13,22]. Consequently, expanding the range of decodable mental tasks has become an important research direction.

Recent studies have explored visual imagery (VI) as an alternative paradigm to MI, offering a broader spectrum of control signals for complex applications. Lee et al. [23] investigated the classification of EEG signals corresponding to visual perception and visual imagery to develop more intuitive BCI systems, demonstrating that preprocessing strategies significantly influence VI classification performance. Kilmarx et al. [24] examined VI as a control strategy by asking participants to observe and imagine four object categories (flower, hammer, face, and scene), achieving promising results in both binary and multi-class tasks. Similarly, Keogh et al. [25] studied the role of mental imagery in enhancing visual working memory, providing insights into how VI can improve BCI interaction. These developments reflect a shift toward more adaptive and user-centered BCI technologies suitable for applications requiring complex and rapid user inputs.

Although PET and fMRI have been utilized in VI-based research [26], their deployment in practical BCI systems is limited due to high cost, immobility, and strict environmental requirements [9,27]. These constraints significantly hinder their applicability in real-world settings [28,29]. As a result, researchers have increasingly adopted EEG for decoding VI tasks. Early EEG-based VI studies reported modest classification performance. Bobrov et al. [30] classified visually imagined faces and houses using Bayesian classifiers, achieving accuracies of 54% and 48%. Kosmyna et al. [31] achieved 55.9% accuracy for two imagined objects using spectrally weighted common spatial patterns, while Esfahani et al. [11] reported 44.6% accuracy for five imagined geometric objects. Costa et al. [32] employed a convolutional neural network (CNN) combined with genetic algorithm optimization to classify four imagined objects, achieving an average accuracy of 60%.

Subsequent studies extended VI-based decoding to imagined letters, words, and speech. Early work on decoding visually observed letters and imagined speech reported accuracies up to 46.6% and 40.1%, respectively [22,33]. Ullah et al. [34] transformed EEG signals using Morlet wavelets and applied a deep CNN to classify 26 imagined English letters, achieving an average accuracy of 95.2%. Datta et al. [35] utilized a multichannel CNN to classify imagined verbs and nouns, achieving 93.8% accuracy across 19 subjects. More recently, Hossain et al. [36] proposed an imagined speech recognition framework for frequently used alphabets and numerals, where a random forest classifier achieved accuracies of 99.38% and 95.39% at coarse and fine levels, respectively. Recent studies further highlight the growing interest in EEG-based decoding of visual information and visual imagery for BCI applications. Wilson et al. [37] reviewed the feasibility of decoding visual information from EEG and emphasized the importance of improving experimental design, data representation, and decoding models for visual BCI tasks. Kilmarx et al. [38] evaluated visual imagery as an EEG-based BCI control strategy and showed that visual imagery can provide classifiable neural signatures for BCI interaction. In addition, recent datasets targeting semantic-concept imagination and visual imagery tasks have been introduced to support the development and benchmarking of VI-based BCI systems [39,40]. These studies indicate that VI-based EEG decoding is an active and emerging research direction, while also highlighting the need for new paradigms that expand the number and diversity of decodable mental commands.

Despite the promising progress in VI-based BCI systems, several critical challenges remain insufficiently addressed. First, most prior studies have focused on relatively simple binary or low-dimensional multi-class tasks, limiting the scalability of VI paradigms for practical assistive control. As the number of classes increases, classification performance often degrades substantially, highlighting the difficulty of reliably decoding fine-grained visual imagery categories from EEG signals. Second, many existing approaches rely on conventional time–frequency transformations or handcrafted features that may not fully capture the joint temporal, spectral, and spatial dynamics of non-stationary EEG signals. Although deep learning models have recently been adopted, their performance is highly dependent on the quality and structure of the input representation, and few studies have systematically investigated high-resolution joint time–frequency–spatial representations tailored specifically for VI decoding [41,42,43,44]. For example, Molina et al. [42] proposed a joint time–frequency–space decorrelation approach for classifying EEG mental tasks, where multichannel EEG signals were spatially decorrelated and then represented in the time–frequency domain for classification. Zhao et al. [44] later proposed WaSF-ConvNet, a deep convolutional network that learns joint space–time–frequency features by combining trainable wavelet-like time–frequency filters with spatial filtering. These studies demonstrated the value of integrating temporal, spectral, and spatial EEG information for BCI decoding. Moreover, existing VI-based frameworks typically decode a single semantic attribute per imagined object (e.g., object category or character identity). The simultaneous decoding of multiple independent attributes from a single imagery task, such as color and direction, remains largely unexplored. This limitation restricts the achievable control dimensionality in current VI-based BCIs and constrains their applicability in complex real-world scenarios. Therefore, there is a clear need for scalable decoding frameworks capable of extracting richer control information from EEG signals while maintaining high accuracy in multi-class settings. Third, color is a fundamental visual attribute and may provide an additional command dimension for VI-based BCI systems. Although EEG-based decoding of imagined color remains less explored than decoding of imagined objects, letters, or directions, previous studies provide a basis for investigating color-related neural information. Hajonides et al. [45] showed that visual color information can be decoded from scalp EEG activity during visual perception. Wu et al. [46] demonstrated that RGB color information can be decoded from EEG/VEP signals using machine learning. In addition, Bannert and Bartels [47] reported that activity patterns in human V4 (hV4) predict behavioral performance during imagery of object color, supporting the involvement of color-related visual regions during internally generated color imagery. These findings motivate the investigation of whether imagined color can be decoded from scalp EEG as part of a VI-based BCI command.

To address these challenges, this study proposes a novel two-phase framework for decoding visually imagined arrows characterized by both color and direction. In the first phase, the Choi–Williams time–frequency distribution (CW-TFD) is employed to construct a high-resolution joint time–frequency–spatial representation (TFSR) of EEG signals. In the second phase, a novel CNN architecture specifically designed for TFSR decoding is introduced. The architecture comprises two sets of layers: the first automatically extracts joint time–frequency–spatial features, while the second encodes the color and direction of an imagined arrow. The proposed framework is evaluated using an EEG dataset recorded from 16 healthy subjects imagining arrows with four directions (up, down, left, right) and four colors (red, green, blue, and white), resulting in 16 distinct classes. Extensive experiments are conducted to assess performance. First, three time–frequency representations Short-Time Fourier Transform (STFT), Continuous Wavelet Transform (CWT), and CW-TFD are compared. Second, the proposed CNN is benchmarked against state-of-the-art architectures, including ResNet-50, VGG-19, ShuffleNet, and GoogleNet. Third, the proposed deep learning framework is compared with conventional classifiers such as Support Vector Machines (SVM), k-Nearest Neighbors (k-NN), Naive Bayes (NB), and Random Forest (RF).

The main contributions of this work are three-fold. First, we introduce a VI-based BCI paradigm for decoding visually imagined arrows represented by 16 combined color–direction classes. Each imagined command is defined by two visual attributes, namely color and direction, thereby expanding the number of decodable mental commands beyond conventional low-dimensional VI tasks. This design directly addresses the scalability limitations of existing VI-based BCIs and demonstrates the feasibility of multi-attribute mental command encoding. Specifically, in this study, scalability refers to increasing the number of decodable visual imagery commands within a subject-specific BCI framework. The proposed paradigm expands the command set by combining two visual attributes, color and direction, while maintaining subject-specific model calibration. Second, we develop a high-resolution joint TFSR based on the CW-TFD, specifically tailored to capture the non-stationary temporal, spectral, and spatial dynamics of EEG signals during visual imagery. Unlike conventional time–frequency approaches, the proposed representation preserves fine-grained joint structure across electrodes, enabling more discriminative feature learning in complex multi-class settings. Third, we propose and systematically validate a novel CNN architecture optimized for decoding TFSR images. The architecture is explicitly designed to hierarchically extract time–frequency features followed by spatial integration, facilitating robust multi-attribute decoding. Through comprehensive benchmarking against alternative time–frequency methods, pre-trained deep networks, and conventional classifiers, we demonstrate that the proposed framework achieves superior performance while maintaining computational feasibility for real-time applications.

The remainder of this paper is organized as follows. Section 2 describes the materials and methods, including the experimental protocol, EEG acquisition and preprocessing procedures, the construction of the proposed TFSR, and the architecture of the proposed CNN model. Section 3 presents the experimental results, including the classification performance, runtime analysis, and comparative evaluations against alternative time–frequency representations, conventional classifiers, and pre-trained deep learning models. Section 4 discusses the implications of the findings, analyzes performance variability, and compares the proposed approach with related work. Finally, Section 5 concludes the paper and outlines directions for future research.

## 2. Materials and Methods

### 2.1. Acquiring and Preprocessing EEG Data

The EEG dataset used in this study was newly collected for the present work and represents a substantial expansion of our preliminary recordings reported in [48]. The preliminary dataset included 10 trials per arrow for each subject, whereas the present dataset includes 80 trials per arrow for each subject.

An experimental protocol was designed to collect EEG signals from a total of 16 healthy volunteers, including 2 females and 14 males, with a mean age of 20.7 years. All participants had normal or corrected-to-normal vision. Prior to participation, each subject provided written informed consent. The experimental procedures complied with the Declaration of Helsinki and were approved by the Research Ethics Committee at the German Jordanian University.

**Figure 1:**
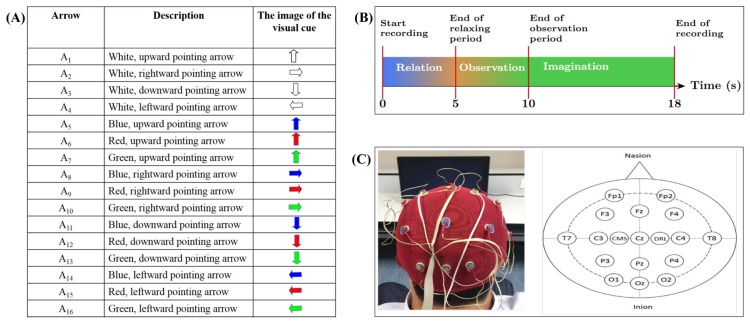
(**A**) Set of 16 visual arrow stimuli (A1–A16) combining direction and color. (**B**) Experimental timeline showing the relaxation, observation, and Visual Imagery phases for each recorded trial. (**C**) The locations of the EEG electrodes.

During the experiment, subjects were seated comfortably on an ergonomically appropriate chair positioned approximately 1 m from a monitor placed directly within their field of view. The monitor automatically displayed the visual stimuli. Each stimulus consisted of an arrow defined by a specific color and direction. Four colors (white, red, green, and blue) and four directions (upward, rightward, downward, and leftward) were used, resulting in a total of 16 distinct arrow stimuli corresponding to 16 different classes. Figure 1A presents the 16 colored arrow stimuli.

During data collection, each subject performed 80 trials per arrow, resulting in a total of 16×16×80=20,480 trials across all participants. Each trial lasted 18 s and consisted of three consecutive phases: relaxation, observation, and imagination. In the relaxation phase, a white screen was displayed for 5 s. During the observation phase, one of the 16 colored arrows was presented for 5 s while the subject attentively observed the stimulus. In the imagination phase, a black screen was displayed for 8 s, and the subject was instructed to close their eyes and vividly imagine the previously observed arrow, including both its color and direction. The timeline of these phases is summarized in Figure 1B.

EEG signals were recorded using the BioSemi ActiveTwo system (BioSemi B.V., Amsterdam, The Netherlands) at a sampling rate of 2048 Hz. Sixteen EEG electrodes were positioned on the scalp according to the international 10–20 electrode placement system [49]. An elastic cap was used to secure the electrodes in place. Figure 1C illustrates the electrode configuration. For noise reduction, signals were referenced to the common mode sense (CMS) and driven right leg (DRL) electrodes during acquisition.

Following data acquisition, the EEG signals underwent several preprocessing steps. First, each trial was bandpass filtered between 0.5 and 32.5 Hz to remove low-frequency drifts and high-frequency noise. Second, signals were re-referenced to the Cz electrode, which was subsequently excluded from further analysis. Third, baseline correction was performed using the last 500 ms of the relaxation phase. Fourth, the signals were downsampled to 256 Hz to reduce computational complexity. Finally, ocular and muscular artifacts were mitigated using the Automatic Artifact Rejection (AAR) toolbox implemented in MATLAB version R2025a [50].

### 2.2. Time–Frequency–Spatial Representation of EEG Signals

EEG signals acquired from different electrodes are analyzed using the Choi–Williams time–frequency distribution (CW-TFD), as summarized in Algorithm 1. The CW-TFD maps one-dimensional EEG signals into a two-dimensional joint time–frequency representation (TFR), capturing the temporal evolution of spectral energy. By computing TFRs for all M=15 electrodes, both temporal-spectral variations and spatial information are preserved. The spatial dimension is defined by the electrode location from which each TFR is derived.
**Algorithm 1** Construction of Time–Frequency–Spatial Representations (TFSRs)  1:**Input:** Preprocessed EEG signals $\left\{x_{m,k}\left(t\right)\right\}$ for m=0,…,M−1 and k=0,…,K−1  2:**Output:** ${\left\{TFSR_{k}\right\}}_{k=0}^{K-1}$  3:**for** k=0 to K−1 **do**  4:      **for** m=0 to M−1 **do**  5:            Compute analytic signal $Z_{m,k}\left(t\right)$  6:            $TFR_{m,k}\left(t,f\right)\leftarrow CW\_TFD\left(Z_{m,k}\left(t\right)\right)$  7:            Convert $TFR_{m,k}$ to grayscale image $G_{m,k}$  8:      **end for**  9:      $TFSR_{k}\leftarrow$ VerticalConcatenate($G_{0,k},\ldots ,G_{M-1,k}$)10:**end for**11:**return** ${\left\{TFSR_{k}\right\}}_{k=0}^{K-1}$

For each electrode $Ch_{m}$, m=0,…,M−1, only the imagination-phase EEG interval from 11 s to 18 s of each trial were used. This interval excluded the full observation phase and the first 1 s of the imagination phase. After downsampling to 256 Hz, the 7 s imagination-only interval was divided into K=7 non-overlapping windows of 256 samples. Each 1 s window was used to construct one TFSR image. Let $x_{m,k}\left(t\right)$ denote the *k*-th segment of electrode $Ch_{m}$. The analytic signal associated with $x_{m,k}\left(t\right)$ is denoted by $Z_{m,k}\left(t\right)$. The CW-TFD of $x_{m,k}\left(t\right)$ is defined as [51,52]:

(1) $${TFR}_{m,k}\left(t,f\right)=\int_{-\infty }^{\infty }\int_{-\infty }^{\infty }\eta_{Z_{m,k}}\left(\phi ,\tau \right)\;\rho \left(\phi ,\tau \right)\;e^{-j2\pi (f\tau +t\phi )}\;d\tau \;d\phi ,$$

where $\eta_{Z_{m,k}}\left(\phi ,\tau \right)$ is the ambiguity function of $Z_{m,k}\left(t\right)$, and

$$\rho \left(\phi ,\tau \right)=\exp\;\left(-\frac{\phi^{2}\tau^{2}}{\beta^{2}}\right)$$

is the exponential kernel that suppresses cross-terms in the CW-TFD. The parameter β controls the smoothing level and is empirically set to β=0.5.

**Figure 2:**
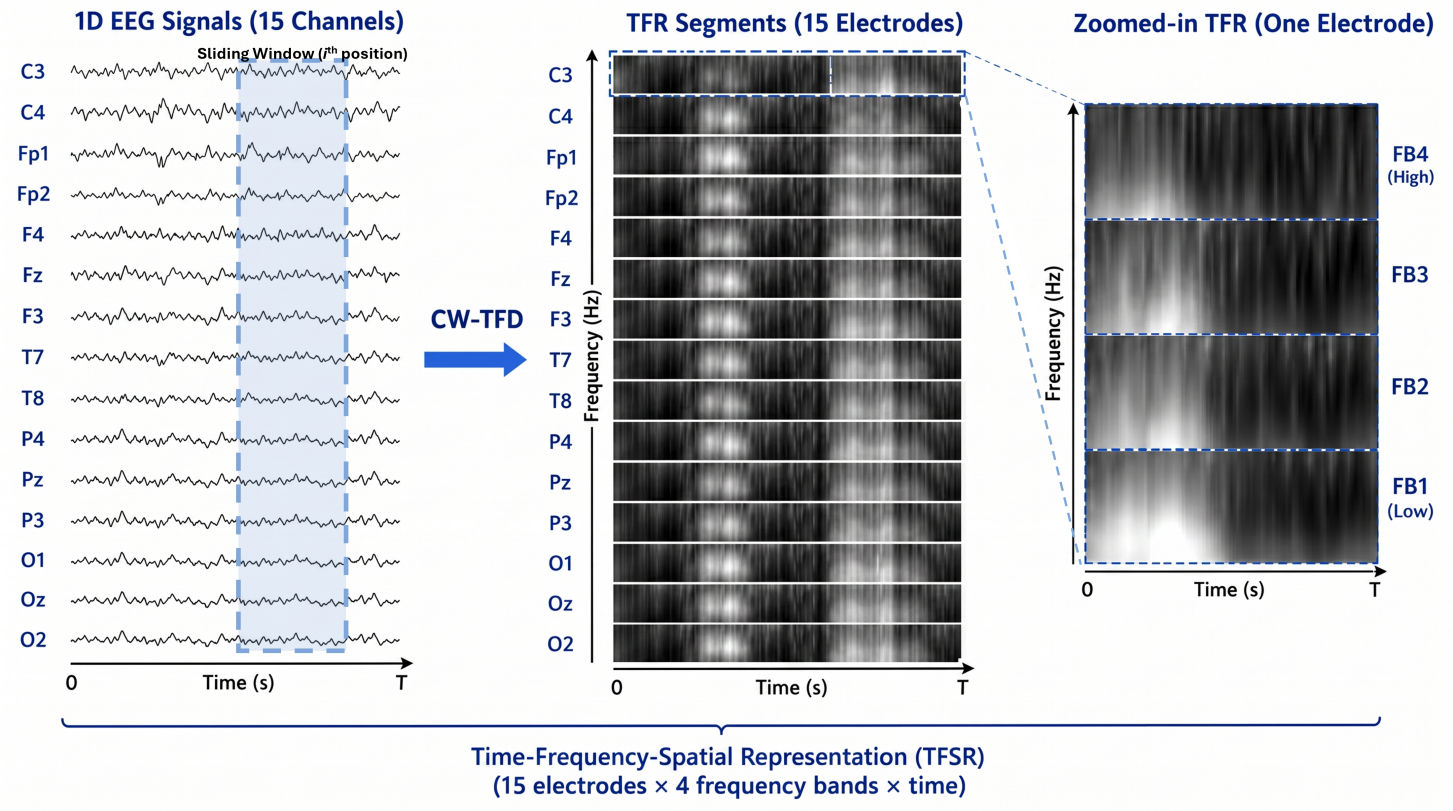
A sample image of the TFSR constructed for the EEG segments at the $i^{th}$ position of the sliding window within the first trial that was recorded for the first subject while imagining $A_{1}$.

Each computed TFR has dimensions 32×256, where the vertical axis corresponds to frequency bins and the horizontal axis corresponds to time samples. The frequency axis spans 0.5–32.5 Hz and is divided into four bands: FB1 [0.5,8.5) Hz, FB2 [8.5,16.5) Hz, FB3 [16.5,24.5) Hz, and FB4 [24.5,32.5) Hz. After computing $TFR_{m,k}$ for all electrodes at window index *k*, each TFR is normalized and converted into a grayscale image (Algorithm 1, line 7). The M=15 grayscale TFR images corresponding to window *k* are vertically concatenated to construct a joint time–frequency–spatial representation (TFSR), denoted as $TFSR_{k}$. The resulting TFSR image has dimensions 480×256 (i.e., 15×32 frequency rows and 256 time columns). Figure 2 illustrates an example TFSR image generated from subject 1 at window position *i* while imagining a white upward-pointing arrow (class A1).

### 2.3. Feature Extraction and Decoding of Visually Imagined Arrows

This section presents the proposed CNN architecture designed to analyze the constructed TFSRs and decode the color and direction of the visually imagined arrows. The CNN consists of two main sets of layers, denoted as Set S1 and Set S2. Set S1 is responsible for hierarchical feature extraction from the temporal, spectral, and spatial information embedded within the input TFSRs, whereas Set S2 performs the final classification.

**Figure 3:**
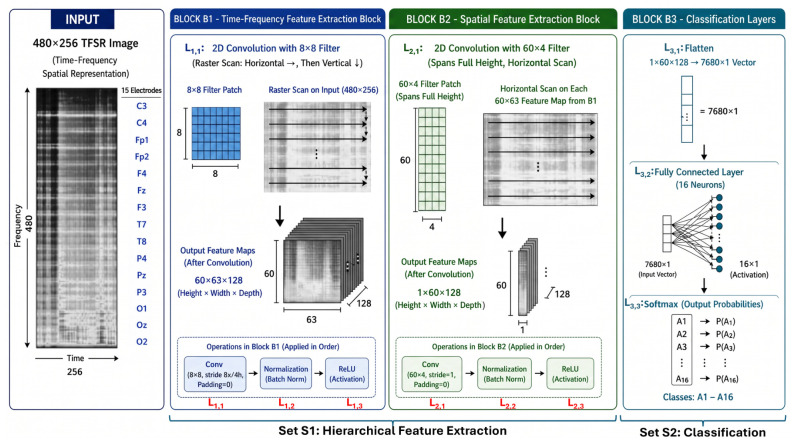
Overview of the proposed CNN architecture showing the grayscale TFSR image used as input and the three cascaded processing blocks: Block 1 (B1)—time–frequency feature extraction layers, Block 2 (B2)—spatial feature extraction layers, and Block 3 (B3)—classification layers.

An overview of the proposed architecture is shown in Figure 3, illustrating the hierarchical organization of the processing blocks B1–B3 and their grouping within Sets S1 and S2.

#### 2.3.1. Set S1: Hierarchical Feature Extraction

Set S1 comprises two processing blocks, B1 and B2. Block B1 focuses on extracting joint time–frequency features independently for each electrode, while Block B2 integrates spatial information across electrodes.

**Block B1 (Time–Frequency Feature Extraction):** Block B1 consists of three layers: $L_{1,1}$, $L_{1,2}$, and $L_{1,3}$ (Figure 3). Layer $L_{1,1}$ applies a set of two-dimensional convolutional filters to the input TFSR image. The filters operate horizontally along the time axis and vertically along the frequency axis within each electrode-specific segment of the TFSR. This operation extracts localized joint time–frequency patterns. Specifically, in layer $L_{1,1}$, the convolutional filters have a size of 8×8 pixels and are applied with a vertical stride of 8 pixels. Since each electrode-specific TFR occupies 32 rows in the vertically concatenated TFSR image, the vertical filter positions remain aligned within each electrode block. Specifically, the filter covers rows 1–8, 9–16, 17–24, and 25–32 within one electrode-specific TFR before moving to the next electrode block. Therefore, no filter in the first convolutional layer overlaps two adjacent electrode-specific TFRs. The outputs are organized into feature maps (FMs), where each FM corresponds to a specific convolutional filter.

Layer $L_{1,2}$ performs feature normalization to stabilize training and improve generalization. The normalized feature maps are then passed to layer $L_{1,3}$, where a rectified linear unit (ReLU) activation function introduces nonlinearity, generating sparsely activated feature maps.

At the output of B1, each FM preserves electrode-wise separation. Specifically, since the TFSR consists of vertically concatenated electrode-specific TFRs (M=15 electrodes), each FM contains 15 sub-maps (SMs), where each SM corresponds to one electrode. Accordingly, Block B1 extracts local time–frequency features within each electrode-specific TFR while preserving electrode-wise separation. Spatial information across electrodes is not mixed in $L_{1,1}$; instead, cross-electrode spatial integration is performed explicitly in Block B2.

**Block B2 (Spatial Feature Extraction):** Block B2, illustrated in Figure 3, also consists of three layers: $L_{2,1}$, $L_{2,2}$, and $L_{2,3}$. Layer $L_{2,1}$ applies two-dimensional convolutional filters across the stacked electrode-specific sub-maps within each feature map, thereby explicitly capturing spatial dependencies among electrodes after within-electrode time–frequency features have been extracted by Block B1. The resulting outputs are referred to as spatial feature maps (SFMs). Layer $L_{2,2}$ performs normalization, and layer $L_{2,3}$ applies a ReLU activation function to introduce nonlinearity. The activated SFMs encode joint time–frequency–spatial features and serve as the input to Set S2.

#### 2.3.2. Set S2: Classification

Set S2 corresponds to Block B3, shown in Figure 3, and performs the final decoding. Layer $L_{3,1}$ flattens the spatial feature maps into a feature vector of dimension 7680×1. Layer $L_{3,2}$ is a fully connected layer with 16 neurons, where each neuron corresponds to one combined color–direction arrow class. Layer $L_{3,3}$ computes class probabilities using a softmax activation function and assigns the input TFSR image to the class with the highest probability, yielding the predicted arrow label $A_{i}$, i∈{1,…,16}. Therefore, the proposed CNN performs single-label 16-class classification of color–direction arrow combinations rather than separate multi-output prediction of color and direction.

### 2.4. Training and Testing of Our Proposed CNN

For each subject, model evaluation was performed using a repeated 10-fold cross-validation protocol with trial-level partitioning. For subjects whose data were recorded across two or three sessions, the recording sessions were mutually exclusive with respect to the imagined object. Specifically, trials corresponding to the same imagined arrow object were not recorded in different sessions; all trials associated with a given object for a given subject were collected within one session only. After data collection, all recorded trials from all sessions of the same subject were combined before cross-validation. The combined trials of each subject were then assigned to 10 class-stratified folds at the level of complete trials. All TFSR images generated from the same EEG trial were assigned to the same fold and inherited the trial label. Therefore, no TFSR images generated from the same trial were present simultaneously in the training and testing subsets. For each cross-validation iteration, the CNN was trained using TFSR images generated from trials in nine folds and tested using TFSR images generated from trials in the held-out fold. The 10-fold cross-validation procedure was repeated 10 times using different random trial-level partitions, and the final performance metrics were computed by averaging the results across all repetitions.

This trial-wise partitioning prevented optimistic bias caused by correlations among TFSR images originating from the same EEG trial. Moreover, the training and testing folds contained only TFSR images generated from the imagination-only EEG interval; no TFSR image derived from the observation phase was included in model training or evaluation.

The parameters of the CNN were optimized using the stochastic gradient descent (SGD) algorithm to minimize the categorical cross-entropy loss function. The model was trained for 50 epochs with a fixed learning rate of 0.01 to ensure stable convergence across repetitions.

All evaluations were conducted in a within-subject setting. Specifically, a separate subject-specific CNN model was trained and evaluated for each participant. Trials from different subjects were not pooled during model training or testing, and no subject-independent or cross-subject evaluation was performed. Therefore, the reported classification results reflect subject-specific decoding performance.

### 2.5. Evaluation Procedures and Metrics

All experiments were implemented in MATLAB and executed on a workstation equipped with 128 GB RAM, an Intel Xeon Gold 6226R (2.9 GHz) processor, and an NVIDIA Quadro RTX 8000 GPU.

#### 2.5.1. Runtime Analysis

The computational efficiency of the proposed framework was evaluated using four time intervals:

- $\tau_{1}$: average time required to construct a single TFSR image.
- $\tau_{2}$: average time required to train the CNN model.
- $\tau_{3}$: average time required to classify a single TFSR image into one of the 16 arrow classes shown in Figure 1.
- $\tau_{4}$: average time required for end-to-end processing, including TFSR construction and classification.

#### 2.5.2. Performance Metrics

The decoding performance was quantified using classification accuracy (Acc) [53] and Cohen’s kappa coefficient (κ) [54,55]. These metrics are defined as follows [53,56]:

(2) $$Acc=\frac{tp+tn}{tp+tn+fp+fn},$$

(3) $$\kappa =\frac{Acc-P_{r}}{1-P_{r}},$$

where tp, tn, fp, and fn denote the numbers of true positives, true negatives, false positives, and false negatives, respectively. The probability of random agreement, $P_{r}$, is computed as

(4) $$P_{r}=\frac{\left(tp+fn)(tp+fp)+(fp+tn)(fn+tn\right)}{{\left(tp+tn+fp+fn\right)}^{2}}.$$

The κ coefficient measures the agreement between predicted and true labels while accounting for agreement occurring by chance. The interpretation of κ values is summarized in Table 1.

#### 2.5.3. Comparative Evaluation

To assess the robustness and generalization capability of the proposed framework, three comparative evaluation procedures were conducted. In the first procedure, the CNN trained on the CW-TFD-based TFSR images was compared with CNN models trained on TFSR images constructed using alternative time–frequency analysis techniques, namely STFT and CWT. In the second procedure, the performance of the proposed CNN architecture was compared with several pre-trained CNN architectures, including ResNet-50, VGG-19, ShuffleNet, and GoogleNet. In the third procedure, handcrafted features extracted from the CW-TFD-based TFSR images were classified using conventional machine learning classifiers, and the results were compared with those of the proposed CNN framework.

#### 2.5.4. Attribute-Specific and Fixed-Direction Evaluation

In addition to the 16-class color–direction decoding task, attribute-specific analyses were conducted to examine whether the proposed framework captured discriminative information related to both visual attributes. For the color-only analysis, the classification task was formulated using four color labels, regardless of arrow direction. For the direction-only analysis, the classification task was formulated using four direction labels, regardless of arrow color. Accuracy and κ were computed separately for each attribute. Since both analyses involved four classes, the chance level was 25%.

To further evaluate whether color decoding was independent of direction information, fixed-direction color analyses were performed. In these analyses, arrow direction was held constant, and color decoding was evaluated separately for downward, upward, rightward, and leftward arrows. For each fixed direction, the classification task involved four color classes, with a chance level of 25%.

For the confusion-matrix analysis, a confusion matrix was first computed separately for each subject because the models were trained and evaluated in a subject-specific manner. Each subject-level confusion matrix was row-normalized, and the normalized matrices were then averaged across subjects to obtain the mean subject-wise confusion matrix.

## 3. Experimental Results

### 3.1. Results of the Proposed CNN Approach

This section presents the classification performance of the proposed model on the 16-class visual imagery task. Results are reported in terms of Acc and κ, along with their corresponding standard deviations (STD), computed per subject across all classes. Moreover, all classification results reported in this section were obtained using the trial-wise repeated cross-validation protocol described in Section 2.4, where all TFSR images derived from the same EEG trial were kept within the same fold. In addition, all results reported in this section were obtained using TFSR images computed exclusively from the imagination phase after excluding the observation phase and the first 1 s of the imagination period.

**Figure 4:**
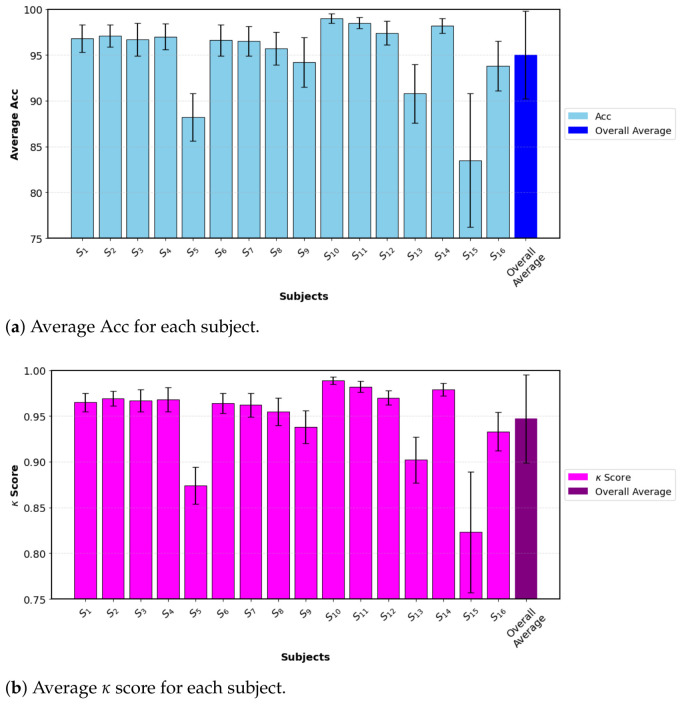
Plots of Acc and κ scores with Standard Deviation (STD) for 16 subjects. Accuracy is displayed in sky-blue and Kappa Score in magenta.

Figure 4 presents the average Acc and κ values (mean ± STD) for each subject across all classes. The proposed model achieved its highest performance for Subject 10, with an accuracy of 99.03±0.52% and κ=0.990±0.003. The lowest performance was observed for Subject 15, with an accuracy of 83.57±7.10% and κ=0.824±0.063. Across all subjects and classes, the overall performance of the model was 95.05±2.03% in terms of Acc and 0.947±0.020 in terms of κ.

**Figure 5:**
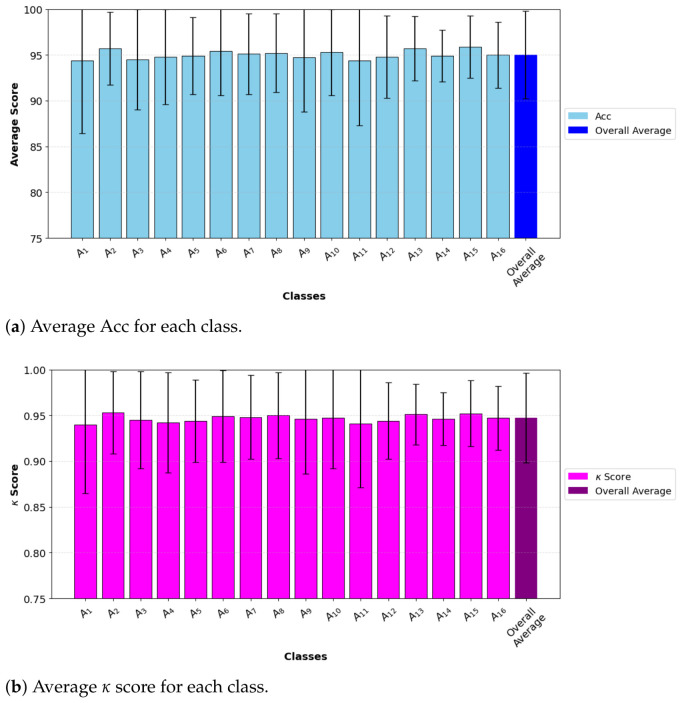
Plots of Acc and κ scores with Standard Deviation (STD) for 16 classes. Accuracy is displayed in sky-blue and Kappa Score in magenta.

Figure 5 illustrates the average Acc and κ values computed for each of the 16 arrow classes over the repeated cross-validation procedure. The results show that the proposed framework consistently achieves performance well above the random decoding rate (6.25% for a 16-class task). Performance variations across classes were minor, indicating stable multi-class decoding capability. The obtained κ values exceed 0.8 for all subjects, corresponding to substantial to almost perfect agreement according to Table 1. This confirms the reliability of the predicted labels relative to ground truth annotations.

### 3.2. Runtime of the Proposed CNN Approach

**Figure 6:**
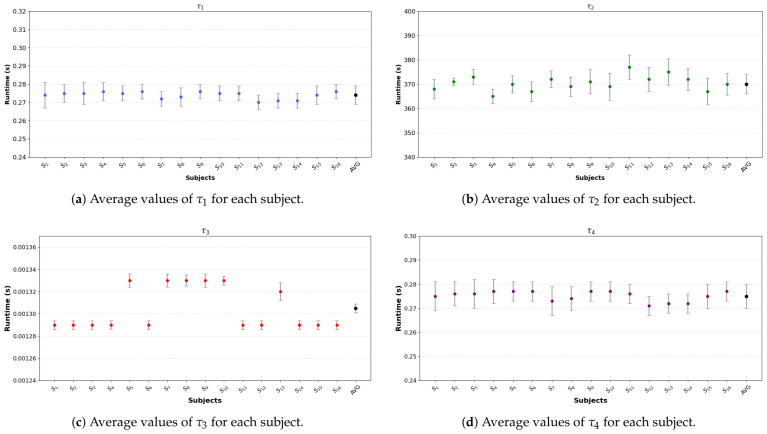
Trends in average runtime and standard deviation across subjects, (**a**) $\tau_{1}$, (**b**) $\tau_{2}$, (**c**) $\tau_{3}$, and (**d**) $\tau_{4}$.

Figure 6 presents the mean and standard deviation of the runtime metrics $\tau_{1}$, $\tau_{2}$, $\tau_{3}$, and $\tau_{4}$ across subjects.

The average runtimes were:

- $\tau_{1}=0.2740\pm 0.0044$ s (TFSR construction),
- $\tau_{2}=370.46\pm 4.09$ s (model training),
- $\tau_{3}=0.00130\pm 0.000004$ s (single-sample classification),
- $\tau_{4}=0.2753\pm 0.0044$ s (end-to-end processing).

The low variance across subjects indicates stable computational performance. Importantly, the sub-second end-to-end processing time ($\tau_{4}$) indicates that, after subject-specific calibration and model training, the proposed framework is computationally feasible for online inference in VI-based BCI applications.

### 3.3. Results Using Different Time–Frequency Analysis (TFA) Techniques

This subsection evaluates the influence of different TFA techniques on classification performance. For the STFT-based TFSR images, a Hanning window of 32 samples without overlap was applied. For the CWT-based TFSR images, a Morse analytic wavelet with symmetry parameter 3 was used. The resulting images were resized to 480×225 (STFT) and 480×256 (CWT) to ensure consistent spatial dimensions and fair comparison.

**Figure 7:**
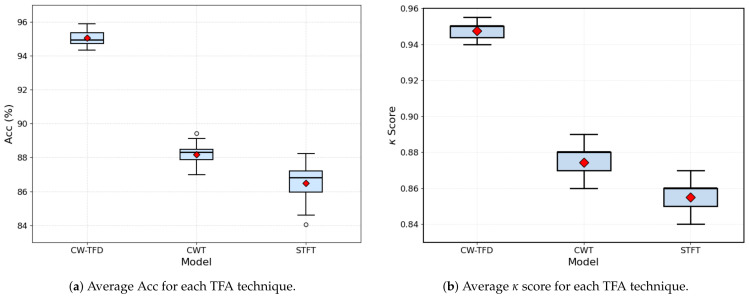
Comparison of classification performance of the proposed CNN model (using CW-TFD-based TFSR images) with alternative TFA techniques, namely CWT and STFT. The boxplots illustrate the distribution of Acc and κ scores for the three representations. The interquartile range (IQR) spans the first quartile (25th percentile), median (50th percentile), and third quartile (75th percentile), while whiskers extend to 1.5 × IQR. Points beyond this range are shown as outliers.

Figure 7 presents the boxplots of Acc and κ values for the three time–frequency approaches. The CWT-based representation achieved 88.18±9.05% accuracy and κ=0.873±0.094, while the STFT-based representation achieved 86.94±10.95% accuracy and κ=0.858. Compared with STFT and CWT, the CW-TFD-based TFSR achieved the highest performance, with an average accuracy of 95.05±2.03% and κ=0.947±0.020. These results demonstrate that the CW-TFD provides a more discriminative TFR for VI-based EEG decoding.

### 3.4. Results Using Handcrafted Features and Conventional Classifiers

This subsection reports classification performance using handcrafted features extracted from the CW-TFD-based TFSR images. A total of 12 time–frequency features were computed per electrode [2,8,57], including logarithmic amplitude sum, median absolute deviation, root mean square, interquartile range, mean, variance, skewness, kurtosis, spectral flatness, spectral flux, normalized Rényi entropy, and energy concentration. Since 15 electrodes were used, the final feature vector had dimensionality 180. The extracted features were classified using SVM (RBF kernel), k-NN (k=7), Naive Bayes (NB), and Random Forest (RF), evaluated under the same repeated cross-validation scheme.

**Figure 8:**
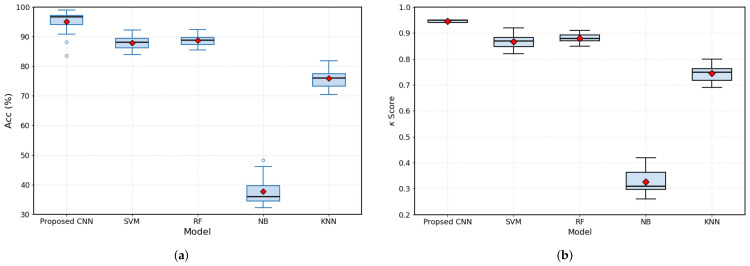
Comparison of classification performance between the proposed CNN model and traditional classifiers using manually crafted features. (**a**) Average Acc for each conventional machine learning model. (**b**) Average κ score for each conventional machine learning model. The boxplots illustrate the distribution of Acc and κ scores for the proposed CNN, SVM, RF, NB, and k-NN classifiers.

Figure 8 shows the distribution of Acc and κ values. The proposed CNN model achieved the best performance with 95.05±2.03% accuracy and κ=0.947±0.020. SVM achieved 87.92±7.85% accuracy and κ=0.87±0.05. RF achieved 88.72% mean accuracy, while NB showed the lowest performance with 37.66% mean accuracy and κ=0.31. These results confirm the superiority of the proposed framework over handcrafted feature-based methods.

### 3.5. Results Using Pre-Trained CNNs

For the pre-trained CNN baselines, each grayscale TFSR image was resized to the input resolution required by the corresponding architecture and replicated across the three input channels to satisfy the input format of ImageNet-pre-trained networks. This conversion did not introduce additional color information, because the same grayscale TFSR image was copied into the red, green, and blue channels. The images were normalized using min–max normalization to [0,1] before training. The original classification layer of each pre-trained CNN was replaced with a new fully connected layer containing 16 neurons, corresponding to the 16 color–direction arrow classes, followed by a softmax classification layer. Moreover, the training was performed using SGD with an initial learning rate of 0.1 for 20 epochs.

**Figure 9:**
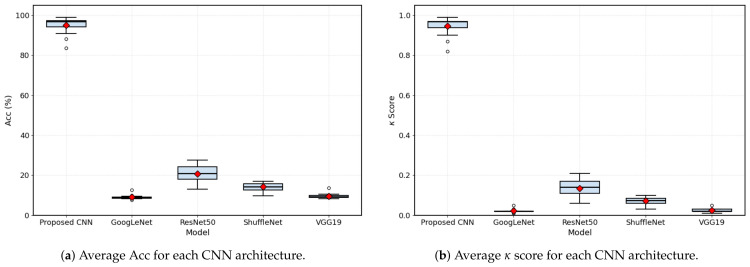
Comparison of classification performance between the proposed CNN model and pre-trained CNN architectures, including GoogLeNet, ResNet-50, ShuffleNet, and VGG-19. The boxplots illustrate the distribution of Acc and κ scores across subjects.

Figure 9 presents the comparative results. The proposed CNN model significantly outperformed all pre-trained models, achieving 95.05% average accuracy and 0.95 average κ. In contrast, GoogleNet and VGG-19 performed near chance level (approximately 9% accuracy), while ResNet-50 and ShuffleNet achieved moderate performance (20.63% and 14.20%, respectively). The κ values for pre-trained models ranged between 0.02 and 0.14, indicating slight to fair agreement.

The results obtained using the four pre-trained CNNs were substantially lower than those achieved by the proposed two-block CNN framework. This performance gap may be attributed to several factors. First, the pre-trained CNNs were originally trained on ImageNet natural images, whereas the target inputs in this study are EEG-derived TFSR images. The source and target domains are therefore highly dissimilar, which can lead to negative transfer, a known phenomenon in transfer learning where knowledge transferred from the source domain negatively affects performance in the target domain [58,59,60,61,62]. Second, the tested pre-trained CNN architectures were not designed to explicitly account for the temporal, spectral, and spatial structure embedded in EEG signals. In contrast, the proposed CNN was specifically designed to extract time–frequency features in the first block and spatial features across electrodes in the second block. We also note that the learning rate of 0.1 used in this baseline transfer-learning configuration is relatively high for fine-tuning pre-trained CNNs and may have contributed to suboptimal convergence. Therefore, these results indicate that, under the evaluated transfer-learning configuration, the proposed task-specific CNN was more effective than the tested ImageNet-pre-trained CNNs for decoding visually imagined colored arrows from EEG TFSR images. They should not be interpreted as evidence that all pre-trained CNNs are inherently unsuitable for EEG-based TFSR decoding.

### 3.6. Attribute-Specific Decoding of Color and Direction

Although the proposed CNN was trained as a single-label 16-class classifier, each class represented a unique color–direction arrow combination. Therefore, additional attribute-specific analyses were conducted to examine whether color and direction were both decodable from the EEG signals.

For the color-only analysis, the task was formulated as a four-class classification problem corresponding to the four arrow colors. For the direction-only analysis, the task was formulated as a four-class classification problem corresponding to the four arrow directions. Since both analyses involved four classes, the chance level was 25%.

The subject-wise results for the color-only and direction-only analyses are presented in Table 2. The proposed framework achieved 94.45±4.27% accuracy and κ=0.926±0.057 for color-only decoding. For direction-only decoding, the framework achieved 94.65±3.91% accuracy and κ=0.929±0.052. These results indicate that both color and direction were highly decodable from the EEG signals. The close performance of the two analyses also suggests that the full 16-class color–direction decoding performance was not dominated by only one attribute.

To examine the error patterns, confusion matrices were generated for color-only and direction-only decoding. Since the models were trained and tested separately for each subject, a confusion matrix was first computed independently for each subject. Each subject-level confusion matrix was then row-normalized, and the normalized matrices were averaged across subjects. The resulting matrices therefore represent the mean subject-wise distribution of predicted labels for each true class. The average row-normalized confusion matrix for color-only decoding is shown in Table 3, while the corresponding matrix for direction-only decoding is shown in Table 4.

### 3.7. Fixed-Direction Color Decoding

To further verify that color decoding was not driven mainly by direction-related information, color classification was evaluated separately within each fixed arrow direction. In each analysis, the direction was held constant and the model classified the imagined color among four classes. Therefore, successful classification in this setting reflected discrimination among imagined colors rather than discrimination among arrow directions. The chance level for each fixed-direction color analysis was 25%.

The subject-wise results of the fixed-direction color decoding analyses are presented in Table 5. Color decoding remained consistently high across all four fixed directions. The proposed framework achieved 96.65±2.95% accuracy and κ=0.955±0.039 for downward arrows, 96.68±3.32% accuracy and κ=0.956±0.044 for upward arrows, 96.98±3.12% accuracy and κ=0.960±0.042 for rightward arrows, and 96.81±1.82% accuracy and κ=0.957±0.024 for leftward arrows. These results demonstrate that imagined color was decodable even when arrow direction was fixed.

## 4. Discussion

In this paper, we presented a novel deep learning framework for decoding visually imagined arrows from EEG signals using TFSRs. The model performance was validated using standard evaluation metrics, demonstrating advances in neurophysiological signal decoding for multi-attribute visual imagery tasks.

The results reported in Section 3 demonstrate the strong decoding capability of the proposed CNN framework in the 16-class classification task (Figure 4 and Figure 5). The highest subject-specific performance reached 99.03±0.52% accuracy with κ=0.990±0.003, indicating almost perfect agreement between predicted and true labels.

Although the proposed CNN achieved high overall performance, variability across subjects was observed. Inter- and intra-subject variability is well documented in EEG-based BCI research and may arise from differences in neural activity patterns, cognitive strategies, attention levels, and neurophysiological characteristics [63,64,65]. It has also been reported that approximately 10–50% of users may exhibit “BCI inefficiency,” characterized by less discriminative neural patterns [63]. Such factors can influence classification performance despite consistent experimental conditions.

The additional attribute-specific analyses provide further insight into the behavior of the proposed framework. Although the CNN was implemented as a single-label 16-class classifier, each class corresponded to a specific color–direction arrow combination. As shown in Table 2, the color-only and direction-only analyses achieved 94.45±4.27% and 94.65±3.91% accuracy, respectively, with corresponding κ values of 0.926±0.057 and 0.929±0.052. The similarity between these values indicates that both visual attributes contributed discriminative information to the classification results. Therefore, the high 16-class accuracy was not mainly driven by only one attribute.

The confusion matrices further support this interpretation. As shown in Table 3, the average diagonal values for color-only decoding ranged from 93.985% to 94.839%. Similarly, Table 4 shows that the average diagonal values for direction-only decoding ranged from 94.422% to 95.009%. In both matrices, the off-diagonal values were small and distributed across the remaining classes, indicating balanced classification performance and no dominant error pattern for either color or direction.

The fixed-direction color analyses provide additional evidence that the framework captured color-related EEG information independently of direction. As reported in Table 5, when arrow direction was held constant, the model continued to decode the imagined color with high accuracy across all four directions, achieving 96.65±2.95%, 96.68±3.32%, 96.98±3.12%, and 96.81±1.82% for downward, upward, rightward, and leftward arrows, respectively. Since direction was fixed in each of these analyses, the obtained performance reflected discrimination among colors only. These results show that the model did not rely mainly on direction-related information.

These findings are important because EEG-based decoding of imagined color is less established than decoding of imagined objects, directions, letters, or words. Prior work has shown that perceived color can be decoded from scalp EEG and that RGB color information can be extracted from EEG/VEP responses [45,46]. In addition, neuroimaging evidence indicates that color-related visual regions, including hV4, are involved during imagery of object color [47]. The present results extend this line of work by demonstrating that imagined color can contribute discriminative information in an EEG-based VI-BCI paradigm involving color–direction arrow commands.

The runtime analysis shown in Figure 6 confirms the practical feasibility of the proposed framework. The average end-to-end processing time ($\tau_{4}$) was 0.2753±0.0044 s per sample, enabling sub-second inference suitable for real-time BCI applications. This real-time feasibility refers to computational processing after a subject-specific model has been trained. It does not imply immediate deployment to unseen users without calibration. The low standard deviation across subjects further demonstrates computational stability independent of user-specific variability.

Comprehensive comparisons were conducted against three categories of alternative approaches: (i) TFSR constructed using STFT and CWT, (ii) handcrafted feature-based pipelines combined with conventional classifiers (SVM, RF, NB, and k-NN), and (iii) pre-trained CNN architectures (GoogLeNet, ResNet50, ShuffleNet, and VGG19). First, when comparing time–frequency representations, the proposed CW-TFD-based TFSR consistently yielded significantly higher decoding performance than both STFT- and CWT-based representations. This finding underscores the importance of high-resolution joint time–frequency analysis in capturing discriminative EEG patterns associated with multi-attribute visual imagery tasks. Second, in comparison with handcrafted feature-based pipelines, the proposed end-to-end CNN significantly outperformed all conventional classifiers, demonstrating the advantage of automatically learning hierarchical time–frequency–spatial features over manually engineered descriptors. Finally, when evaluated against pre-trained CNN architectures originally designed for natural image recognition, the proposed model achieved substantially superior performance, indicating that generic image-based architectures are not well suited for structured EEG TFSR inputs without task-specific architectural adaptation. The lower performance of the pre-trained CNN baselines should be interpreted in the context of transfer learning. ImageNet-pre-trained CNNs learn features from natural images, whereas EEG TFSR images represent structured temporal, spectral, and spatial neural activity. This mismatch between the source and target domains may result in negative transfer [58,59,60,61,62]. Moreover, standard pre-trained CNN architectures do not explicitly model the organization of EEG TFSR images, where frequency bands, time samples, and electrode-specific spatial information have defined physiological meaning. The proposed two-block CNN was designed around this structure, which likely contributed to its higher decoding performance. Nevertheless, this comparison does not rule out the possibility that alternative fine-tuning strategies, lower learning rates, domain-specific pretraining, or self-supervised EEG pretraining could improve transfer-learning performance for EEG TFSR decoding. Collectively, these results demonstrate that the proposed framework consistently outperforms alternative representation strategies and competing classification models, validating the effectiveness of its tailored representation design and specialized CNN architecture for EEG-based visual imagery decoding.

To statistically compare the classification performance across the evaluated approaches, the Friedman test was employed. The Friedman test is a non-parametric alternative to repeated-measures ANOVA and was selected because classification accuracies were computed for the same 16 subjects under multiple related conditions, thereby accounting for within-subject dependency without assuming normality.

First, the Friedman test was applied to compare the three TFSRs (STFT, CWT, and CW-TFD). The results reveal a statistically significant difference among the three methods, $\chi^{2}\left(2\right)=32.0$, p<0.001. The effect size, measured using Kendall’s *W*, was 1.0, indicating a perfectly consistent ranking across subjects. Post hoc Wilcoxon signed-rank tests with Bonferroni correction (α=0.0167) showed that the CW-TFD-based TFSR significantly outperformed both CWT and STFT (p<0.001 in both comparisons), and that CWT significantly outperformed STFT (p<0.001). These findings confirm the superiority of the CW-TFD-based representation for multi-class visual imagery decoding.

Second, when comparing the proposed CNN with handcrafted feature-based pipelines combined with conventional classifiers (SVM, RF, NB, and k-NN), the Friedman test revealed a statistically significant difference among the five classifiers, $\chi^{2}\left(4\right)=59.05$, p<0.001. The corresponding Kendall’s *W* was 0.923, indicating a very strong and consistent effect across subjects. Post hoc Wilcoxon signed-rank tests with Bonferroni correction (α=0.0125) demonstrated that the proposed CNN significantly outperformed SVM ($p=5.80\times 10^{-4}$), RF ($p=5.80\times 10^{-4}$), NB (p<0.001), and k-NN (p<0.001). These results highlight the advantage of end-to-end hierarchical feature learning over manually engineered feature extraction.

Finally, the Friedman test was conducted to compare the proposed CNN with pre-trained CNN architectures (GoogLeNet, ResNet50, ShuffleNet, and VGG19). The analysis revealed a statistically significant difference among the five models, $\chi^{2}\left(4\right)=61.60$, p<0.001, with Kendall’s W=0.963, indicating an extremely strong and consistent effect across subjects. Post hoc Wilcoxon signed-rank tests with Bonferroni correction (α=0.0125) confirmed that the proposed CNN significantly outperformed all pre-trained models (all p<0.001). This demonstrates that generic image-based CNN architectures are not well suited for structured EEG TFSR inputs without task-specific architectural adaptation.

Overall, the statistical analyses consistently demonstrate that the proposed CNN combined with the CW-TFD-based TFSR achieves significantly superior decoding performance across all evaluated alternatives, with strong and consistent effects observed for all subjects.

Table 6 provides a structured comparison between the proposed framework and previously reported approaches in terms of the number of classes, trials, subjects, and achieved accuracy. Compared with earlier visual imagery and object decoding studies, the proposed framework demonstrates competitive and, in several cases, superior performance despite addressing a relatively complex 16-class multi-attribute task. For example, Korik et al. [66] reported 30% accuracy for five 3D objects using regularized LDA, while Costa et al. [32] achieved 60% accuracy for four objects using a CNN-based approach. Although Le [67] reported 89.3% accuracy for a two-class scene classification task, the reduced class space makes the problem substantially less complex than the present 16-class decoding scenario. Studies such as Ullah et al. [34] and Hossain et al. [36] reported accuracies above 95% for alphabet and numeral recognition tasks; however, these paradigms differ in structure and class composition from the multi-attribute arrow decoding task considered in the current study. Alazrai et al. [48] investigated a preliminary version of the 16-arrow dataset containing only 10 trials per arrow for each subject. Their approach used CW-TFD-based handcrafted features combined with extensive channel and feature selection procedures and an SVM classifier, achieving 92.68% accuracy. In contrast, the present study used a substantially expanded dataset with 80 trials per arrow for each subject and achieved 95.05% accuracy using the proposed CW-TFD-based TFSR representation and task-specific CNN. Because the two studies differ in dataset size and learning strategy, this comparison is interpreted as contextual rather than a strict head-to-head comparison. Nevertheless, the result supports the effectiveness of the proposed hierarchical CNN framework for decoding visually imagined color–direction arrow combinations. This consistent improvement highlights the advantage of the proposed end-to-end hierarchical learning strategy and the effectiveness of integrating CW-TFD-based TFSR with a tailored CNN architecture.

Compared with previous joint time–frequency–space EEG decoding methods, the proposed framework uses a different representation and targets a different BCI paradigm. Molina et al. [42] relied on joint decorrelation and time–frequency classification of EEG mental tasks, whereas Zhao et al. [44] learned wavelet-based time–frequency filters and spatial filters in an end-to-end ConvNet. In contrast, our approach first constructs CW-TFD-based TFSR images and then applies a CNN architecture tailored to the structure of these images. This design allows the model to extract localized time–frequency features within each electrode before explicitly integrating spatial information across electrodes. The framework was evaluated on a 16-class visual imagery task involving imagined arrows with different colors and directions, which differs from the motor-imagery and mental-task paradigms considered in the studies depicted in [42,44].

A limitation of the present study is that all experiments were conducted using a within-subject evaluation protocol. A separate model was trained and tested for each participant, and no cross-subject or subject-independent evaluation was performed. Therefore, the reported results demonstrate the feasibility of subject-specific decoding of visually imagined color–direction arrow combinations, but they do not establish generalization to unseen users. Future work should evaluate cross-subject generalization, transfer learning, and calibration-reduction strategies to determine whether the proposed framework can be adapted efficiently to new users. Another limitation of this study is that the proposed framework was evaluated using a single newly collected EEG dataset. Although additional public EEG datasets exist for visual imagery tasks involving objects, figures, animals, semantic concepts, and letters, we did not identify a publicly available EEG dataset with the same paradigm of visually imagined arrows defined by both direction and color. Therefore, the present study is, to the best of our knowledge, among the first to explore this specific VI-BCI decoding direction.

Overall, the comparative analysis indicates that the proposed framework achieves state-of-the-art performance for multi-class visual imagery decoding while maintaining robustness across subjects.

## 5. Conclusions

In this study, a deep learning framework was developed for decoding visually imagined arrows from EEG signals using a TFSR constructed via the CW-TFD. The experimental results demonstrate the effectiveness of the proposed TFSR-based approach in capturing discriminative patterns associated with multi-attribute visual imagery tasks.

The proposed CNN achieved an average Acc of 95.05% with κ of 0.947, outperforming conventional classifiers and pre-trained CNN models. In particular, representations derived from CW-TFD consistently yielded superior performance compared with those obtained using STFT and CWT, confirming the importance of high-resolution time–frequency analysis in EEG decoding tasks. The runtime analysis further confirmed the feasibility of the proposed approach for real-time applications, with sub-second inference performance and stable computational behavior across subjects. The findings should be interpreted within the scope of the within-subject evaluation design used in this study. The reported results demonstrate subject-specific decoding performance, where a separate model was trained and evaluated for each participant. Cross-subject generalization and deployment without user-specific calibration were not evaluated in the present work.

Overall, the proposed framework supports accurate subject-specific decoding of visually imagined colored arrows from EEG-derived TFSR images, while further evaluation is required to determine its performance in subject-independent and reduced-calibration settings. Therefore, a possible future research direction is to evaluate the proposed framework on additional public EEG datasets once compatible visual imagery datasets become available. In addition, future studies may also examine the generalizability of the framework to related VI paradigms and benchmark its performance against state-of-the-art methods using standardized evaluation protocols.

## Figures and Tables

**Table 1:** Interpretation of the κ values [56].

Range	Interpretation
κ≤0	No agreement
0<κ≤0.2	Slight agreement
0.2<κ≤0.4	Fair agreement
0.4<κ≤0.6	Moderate agreement
0.6<κ≤0.8	Substantial agreement
0.8<κ≤1	Almost perfect agreement

**Table 2:** Subject-wise color-only and direction-only decoding results.

Subject	Color Acc. (%)	Color κ	Direction Acc. (%)	Direction κ
S1	95.29	0.937	96.20	0.949
S2	96.99	0.960	96.73	0.956
S3	96.88	0.958	96.34	0.951
S4	96.46	0.953	96.59	0.954
S5	86.64	0.822	88.18	0.842
S6	96.69	0.956	95.93	0.946
S7	95.43	0.939	95.38	0.938
S8	94.72	0.930	94.72	0.930
S9	93.01	0.907	93.47	0.913
S10	98.68	0.982	98.63	0.982
S11	98.19	0.976	98.14	0.975
S12	96.92	0.959	97.07	0.961
S13	91.31	0.884	91.34	0.885
S14	97.61	0.968	98.15	0.975
S15	83.17	0.776	84.04	0.787
S16	93.18	0.909	93.42	0.912
Average	94.45 ± 4.27	0.926 ± 0.057	94.65 ± 3.91	0.929 ± 0.052

**Table 3:** Mean row-normalized confusion matrix for color-only decoding (%).

**True\Predicted**	Blue	Green	Red	White
Blue	93.985	1.862	2.213	1.940
Green	1.900	94.495	1.897	1.707
Red	1.805	1.869	94.476	1.851
White	1.699	1.722	1.741	94.839

**Table 4:** Mean row-normalized confusion matrix for direction-only decoding (%).

**True\Predicted**	Downward	Leftward	Rightward	Upward
Downward	94.440	1.828	1.824	1.908
Leftward	1.918	94.710	1.512	1.859
Rightward	1.559	1.623	95.009	1.809
Upward	1.770	1.969	1.839	94.422

**Table 5:** Subject-wise fixed-direction color decoding results.

Subject	Downward Acc. ± STD	Downward κ± STD	Upward Acc. ± STD	Upward κ± STD	Rightward Acc. ± STD	Rightward κ± STD	Leftward Acc. ± STD	Leftward κ± STD
S1	98.51 ± 0.36	0.980 ± 0.001	98.52 ± 0.37	0.980 ± 0.004	97.56 ± 0.14	0.968 ± 0.009	97.99 ± 0.68	0.973 ± 0.008
S2	98.66 ± 0.80	0.982 ± 0.006	98.88 ± 0.57	0.985 ± 0.004	97.47 ± 1.27	0.966 ± 0.004	97.41 ± 0.66	0.965 ± 0.003
S3	97.70 ± 0.98	0.969 ± 0.008	98.38 ± 0.94	0.978 ± 0.010	97.80 ± 0.87	0.971 ± 0.004	98.01 ± 0.63	0.973 ± 0.010
S4	97.87 ± 0.86	0.972 ± 0.009	98.09 ± 0.63	0.974 ± 0.002	98.37 ± 0.91	0.978 ± 0.008	96.81 ± 1.31	0.957 ± 0.014
S5	93.01 ± 1.29	0.907 ± 0.012	92.49 ± 1.83	0.900 ± 0.018	93.08 ± 1.29	0.908 ± 0.012	93.99 ± 1.91	0.920 ± 0.017
S6	97.49 ± 0.96	0.966 ± 0.005	97.20 ± 1.49	0.963 ± 0.009	98.44 ± 0.71	0.979 ± 0.002	97.62 ± 0.92	0.968 ± 0.008
S7	97.47 ± 1.14	0.966 ± 0.014	97.77 ± 1.48	0.970 ± 0.007	98.22 ± 0.21	0.976 ± 0.006	96.65 ± 1.61	0.955 ± 0.016
S8	96.08 ± 1.14	0.948 ± 0.010	97.02 ± 0.56	0.960 ± 0.006	97.84 ± 0.44	0.971 ± 0.003	96.58 ± 1.96	0.954 ± 0.009
S9	98.59 ± 0.49	0.981 ± 0.003	94.94 ± 1.46	0.933 ± 0.016	94.49 ± 1.69	0.927 ± 0.016	97.40 ± 1.32	0.965 ± 0.011
S10	99.04 ± 0.12	0.987 ± 0.003	99.78 ± 0.24	0.997 ± 0.002	99.63 ± 0.32	0.995 ± 0.002	98.96 ± 0.54	0.986 ± 0.005
S11	99.26 ± 0.61	0.990 ± 0.002	99.11 ± 0.73	0.988 ± 0.004	99.33 ± 0.74	0.991 ± 0.006	98.51 ± 0.66	0.980 ± 0.005
S12	97.19 ± 1.61	0.962 ± 0.013	97.84 ± 0.49	0.971 ± 0.007	99.03 ± 0.68	0.987 ± 0.004	97.85 ± 0.91	0.971 ± 0.006
S13	94.12 ± 2.17	0.922 ± 0.020	94.27 ± 1.58	0.924 ± 0.013	97.58 ± 0.65	0.968 ± 0.010	94.56 ± 1.08	0.928 ± 0.005
S14	98.15 ± 1.77	0.975 ± 0.011	99.41 ± 0.36	0.992 ± 0.003	98.21 ± 1.05	0.976 ± 0.007	98.97 ± 0.54	0.986 ± 0.004
S15	87.80 ± 2.53	0.837 ± 0.033	86.68 ± 2.75	0.822 ± 0.022	87.05 ± 3.87	0.827 ± 0.041	93.01 ± 1.86	0.907 ± 0.022
S16	95.39 ± 2.15	0.938 ± 0.024	96.43 ± 0.76	0.952 ± 0.008	97.54 ± 0.77	0.967 ± 0.006	94.60 ± 2.19	0.928 ± 0.018
Average	96.65 ± 2.95	0.955 ± 0.039	96.68 ± 3.32	0.956 ± 0.044	96.98 ± 3.12	0.960 ± 0.042	96.81 ± 1.82	0.957 ± 0.024

**Table 6:** A comparison with previous approaches.

Reference	Num. of Objects	Trials	Algorithm	Subjects	Imagine Arrows	Acc%
Korik et al. (2024) [66]	5 3D primitive object	360	Regularized Linear Discriminant Analysis	10	No	30%
Le (2024) [67]	2 scences	100	1D CNN	20	No	89.3%
Hossain et al. (2024) [36]	20 Alphabets and numerals	No	KNN, RF, SVM	30	No	95.39%
Ullah et al. (2021) [34]	26 letters	NA	Deep CNN	10	No	95.2%
Costa et al. (2022) [32]	4 objects	40	CNN, GA	4	No	60%
Alazrai et al. (2020) [48]	16 arrows	10	SVM	16	Yes	92.68%
**Our Proposed CNN**	**16 arrows**	80	**CNN + TFSR**	**16**	**Yes**	**95.05%**

## Data Availability

The raw data supporting the conclusions of this article will be made available by the authors on request.
